# Mesencephalic Astrocyte-Derived Neurotrophic Factor Is Involved in Inflammation by Negatively Regulating the NF-κB Pathway

**DOI:** 10.1038/srep08133

**Published:** 2015-02-02

**Authors:** Lijian Chen, Lijie Feng, Xia Wang, Jian Du, Ying Chen, Wen Yang, Chengyue Zhou, Li Cheng, Yujun Shen, Shengyun Fang, Jun Li, Yuxian Shen

**Affiliations:** 1 School of Pharmacy, Anhui Medical University, Hefei 230032, Anhui, China; 2 School of Basic Medical Sciences, Anhui Medical University, Hefei 230032, Anhui, China; 3 Institute of Biopharmaceuticals, Anhui Medical University, Hefei 230032, Anhui, China; 4 Department of Anesthesiology of the First Affiliated Hospital, Anhui Medical University, Hefei 230022, Anhui, China; 5 Center for Biomedical Engineering and Technology, University of Maryland, Baltimore, MD 21201, USA

## Abstract

Inflammation can cause endoplasmic reticulum (ER) stress and therefore activates the unfolded protein response (UPR). ER stress and the consequent UPR have the potential to activate NF-κB. However, the factors mediating the crosstalk between ER stress and the NF-κB pathway remain unclear. Here, we determined that ER stress inducible protein Mesencephalic Astrocyte-derived Neurotrophic Factor (MANF) was up-regulated in autoimmune diseases and inflammatory disease models. Inflammation caused MANF to relocalize to the nuclei. MANF interacted with the DNA binding domain of p65 through its C-terminal SAP-like domain in the nuclei under the condition of inflammation or ER stress. MANF consequently inhibited p65-mediated transcriptional activation by interfering with the binding of p65 to its target genes promoters. Consistently, MANF suppressed the expressions of NF-κB-dependent target genes and the proliferation of inflammatory synoviocytes. These findings suggest that MANF may be a negative regulator of inflammation and mediate the crosstalk between the NF-κB pathway and ER stress.

The endoplasmic reticulum (ER) mediates a specific set of intracellular signaling pathways in response to the accumulation of unfolded or misfolded proteins, which is called the unfolded protein response (UPR). Inflammation can cause ER stress and therefore activates its consequent UPR. In mammalian cells, the main UPR signaling cascades are initiated by three ER-localized protein sensors: inositol-requiring 1α (IRE1α), double-stranded RNA-dependent protein kinase (PKR)-like ER kinase (PERK), and activating transcription factor 6 (ATF6). When activated, all three sensors of the UPR participate in regulating inflammatory processes^1^,^2^. ER stress-induced UPR signaling play an important role in the pathogenesis and progression of autoimmune diseases and other inflammatory diseases^3^,^4^,^5^.

NF-κB is a key transcriptional regulator that has a central role at the onset of inflammation following IκB degradation^6^,^7^. The UPR signaling pathway and NF-κB are interconnected through all three branches of the UPR. ER-resident IRE1α is required for NF-κB activation through the TRAF2-mediated formation of a complex between IRE1α and IKK, which causes IκB degradation^8^,^9^. Activated PERK-eIF2a causes translational arrest, which leads to a decrease in IκB protein level and a consequent increase in the ratio of NF-κB to IκB. This ratio change in the ratio causes the release of NF-κB protein, which then performs its pro-inflammatory transcriptional role in the nucleus^10^. The ATF6 branch of the UPR can also activate NF-κB. Loss of the glucose-regulated ER stress protein Grp78 (BiP) by subtilase cytotoxin (SubAB), a protease that selectively degrades Grp78, leads to transient phosphorylation of Akt and consequent activation of NF-κB through the ATF6 branch of the UPR^11^,^12^. Recent reports have suggested that ER stress induced activation of NF-κB in the early phase, whereas in the later phase, consequent UPR inhibited NF-κB signaling^13^,^14^,^15^,^16^. However, the mechanisms underlying the anti-inflammatory potential of ER stress have not been elucidated.

Mesencephalic astrocyte-derived neurotrophic factor (MANF; also known as ARMET) belongs to the fourth family of neurotrophic factors. MANF protects neurons and alleviates the Parkinson's disease-like symptoms in rat 6-hydroxydopamine model. In non-neuronal cells, MANF has also been identified as a secretion protein induced by ER stress that protects against various forms of ER stress-induced damage^17^,^18^,^19^,^20^. In this study, we detected MANF expression in the peripheral white blood cells (PWBC) isolated from the patients with rheumatoid arthritis (RA) or systemic lupus erythematosus (SLE) and from rabbits with antigen-induced arthritis (AIA). The role of MANF involved in inflammation was also investigated by using primarily cultured fibroblast-like synoviocytes (FLS). Our data demonstrated that MANF functioned as an inhibitor of the NF-κB signaling pathway by blocking the binding of p65 to the promoter of its target genes. Consistently, MANF suppressed the expressions of NF-κB dependent genes. MANF knockdown enhanced the proliferation of inflammatory synoviocytes. Therefore, this study suggests that MANF may be a novel negative regulator of inflammation by interacting with p65.

## Results

### Up-regulation of MANF in inflammatory diseases

**Figure 1:**
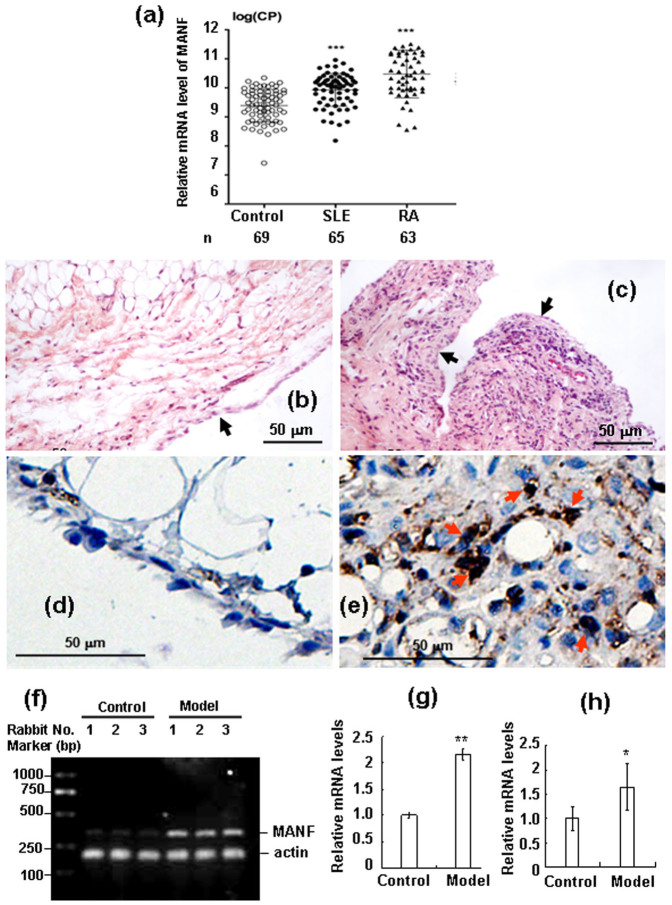
Induction of MANF in inflammatory diseases and rabbit antigen-induced arthritis. (a) The levels of MANF mRNA in SLE (n = 65) and RA (n = 63) patients were detected by real-time qPCR. The data are represented as the mean ± SD. *** P<0.0001, compared with the controls (n = 69). The synovial tissues of normal (b) and antigen-induced arthritis (AIA) rabbit (c) were stained by HE. The arrow in panel (b) shows the lining cell layer of the synovium. The arrows in panel (c) show the proliferative synovium. MANF expression in normal (d) and AIA rabbit synovial tissue (e) was detected by immunohistochemistry technique with an anti-MANF antibody. The experiments in panel b-e were repeated for at least three times. Scale bar = 50 µm. (f) The mRNA levels of MANF were determined by RT-PCR in the synovial tissues from normal and AIA rabbits. (g) The quantitative data of panel (f). The values are expressed as the mean ± SD of three individuals. ** P = 0.002, compared with the controls. (h) The levels of MANF mRNA in PWBC from AIA rabbits were detected by real-time qPCR. The data were represented as the mean ± SD. n = 6, * P = 0.022, compared with the normal control.

We detected MANF expression in PWBC from healthy individuals and RA and SLE patients using the absolute quantitative real-time PCR method. Compared with the healthy controls, MANF was dramatically up-regulated in these patients (Fig. 1a), which suggests that MANF might be involved in the pathogenesis of inflammatory diseases. To confirm this result, we established rabbit arthritis model with methylated bovine serum albumin. The mRNA expressions of MANF in PWBC and synovium were detected by real-time qPCR and RT-PCR, respectively. We found that MANF mRNA was remarkably increased both in the PWBC (Fig. 1h) and in the synovial tissues of AIA rabbits (Fig. 1f–g), compared with that from the sham controls. Furthermore, the typical MANF-positive cells were found in the severe inflammatory regions (Fig. 1e, indicated by arrows), where HE staining showed marked synovial thickening and inflammatory cell infiltration (Fig. 1c, indicated by arrows). These results indicate that MANF is highly associated with arthritis or inflammation.

### Induction of MANF in the fibroblast-like synoviocytes (FLS) of AIA

**Figure 2:**
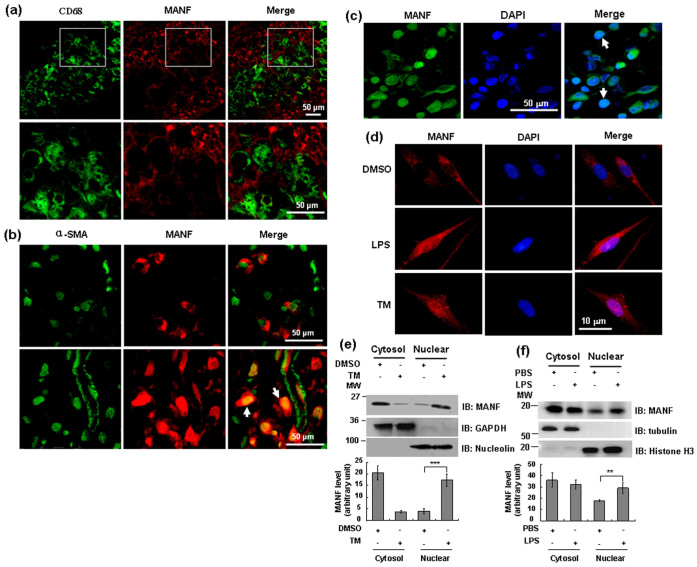
MANF was specifically induced in the FLS of AIA rabbit synovial tissue and relocalized to the nuclei under ER stress. (a) Double-labeled immunofluorescence of CD68 (green) and MANF (red) in the synovial tissues of AIA rabbits. (b) Double-labeled immunofluorescence of a-SMA (green) and MANF (red) in the synovial tissues of AIA rabbits. CD68 and a-SMA were used as the markers of the macrophage-like synoviocytes (MLS) and the fibroblast-like synoviocytes (FLS), respectively. Scale bar = 50 µm. (c) MANF localization in the nuclei in some FLS in the inflammatory synovial tissue. The synovium of AIA rabbits was stained with anti-MANF (green) antibody. DAPI was used to stain the nuclei (blue). Scale bar = 50 µm. (d) MANF was relocalized to FLS nuclei after treatment with LPS and tunicamycin (TM). The FLS isolated from the synovium of normal rabbits were treated with LPS (10 μg/ml) for 48 hrs or TM (2.5 μg/ml) for 16 hrs. Then, the cells were stained with anti-MANF antibody (red) and DAPI (blue). Scale bar = 10 µm. The experiments in panel a-d were repeated for at least three times. (e) TM-induced nuclear localization of MANF in FLS was detected by Western blotting. The FLS isolated from AIA rabbits were treated with TM (2.5 μg/ml) for 16 hrs. The cytosolic and nuclear fractions were subjected to Western blotting with anti-MANF antibody. GAPDH and nucleolin were used as the markers of cytosol and nucleus, respectively. The values are expressed as the mean ± SD of three individuals. *** P<0.0001, compared with DMSO control. (f) LPS-induced MANF nuclear localization in FLS was detected by Western blotting. The FLS were treated with LPS (10 μg/ml) for 48 hrs. Western blotting was used to detect MANF expression in the cytoplasm and nucleus. Tubulin and histone H3 were used as the markers of cytosol and nucleus, respectively. The values are expressed as the mean ± SD of three individuals. ** P = 0.001, compared with PBS control.

FLS and macrophage-like synoviocytes (MLS) are the main inflammatory cell types in the synovium that have been proposed to trigger synovial inflammation. To investigate the subpopulations of MANF-positive cells, we labeled the cells with the antibodies against α-smooth muscle actin (α-SMA, a marker of FLS) and CD68 (a marker of activated macrophages). We found that the positive immunoreactivity of MANF was mainly present in α-SMA-positive FLS (Fig. 2b), and very little was found in CD68-positive MLS (Fig. 2a), suggesting that MANF was mainly induced in the FLS of AIA. In the α-SMA-positive FLS, we found MANF presented both in the cytoplasm ((Fig. 2b, upper panel) and in the nuclei (Fig. 2b, lower panel, as indicated by arrows).

### Inflammation and ER stress induces MANF to re-localize to nuclei in the FLS

In some FLS in the synovium of AIA rabbits, MANF appeared to be in the nuclei (Fig. 2b, lower panel, as indicated by arrows), where it co-localized with α-SMA. With regard to the subcellular localization, we found that MANF overlapped with DAPI in some FLS in the inflammatory synovium (Fig. 2c, indicated by arrows). To verify whether inflammation or ER stress induced MANF re-localization to the nuclei, the primarily cultured FLS isolated from normal synovium were treated with lipopolysaccharide (LPS) or tunicamycin (TM). DMSO was used as vehicle control. The results showed that MANF was mainly presented in the cytoplasm in control cells (Fig. 2d, upper panel). However, when the cells were treated with LPS (10 μg/ml) for 48 hrs or TM (2.5 μg/ml) for 16 hrs, the nuclear MANF was increased (Fig. 2d, middle and lower panel, respectively). Meanwhile, Western blotting also showed that nuclear MANF was increased following TM treatment (Fig. 2e). LPS treatment also increased the nuclear expression of MANF in FLS (Fig. 2f). Our data showed that MANF was effectively located to nucleus in presence of tunicamycin as compared to LPS. These results indicate that either inflammation or ER stress induces MANF nuclear translocation.

### Identification of MANF as a p65-interacting protein

**Figure 3:**
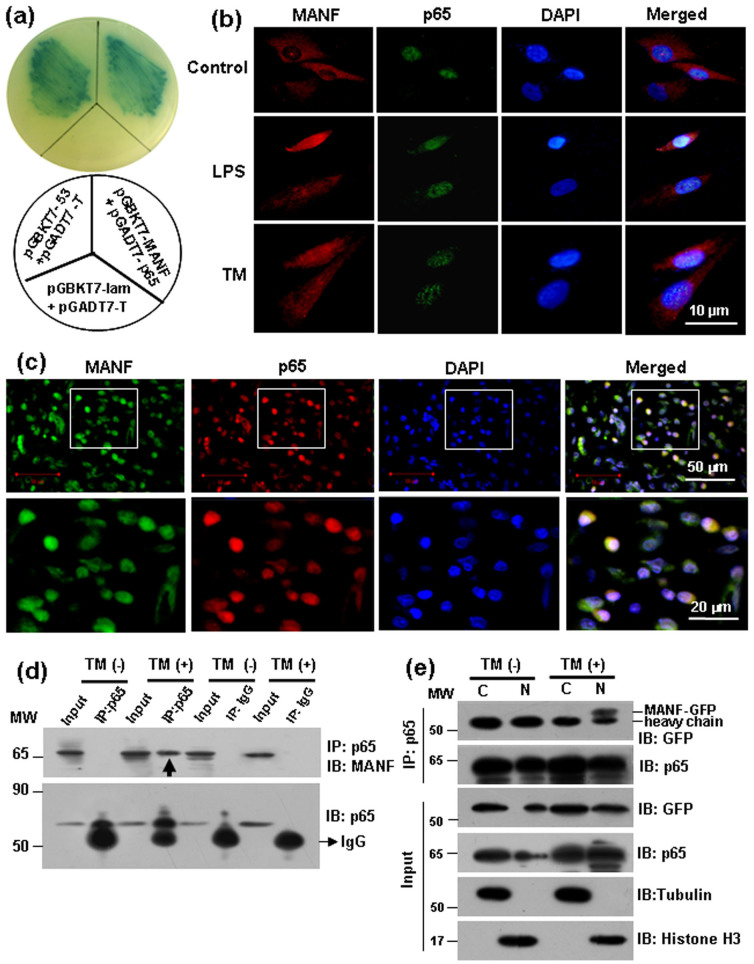
Identification of MANF as a p65-interacting protein in the nuclei. (a) Identification of the interaction of MANF and p65 by a yeast two-hybrid assay. (b) Co-localization of MANF and p65 in the primarily cultured FLS. The FLS isolated from normal rabbits were treated with LPS (10 μg/ml) for 48 hrs or TM (2.5 μg/ml) for 16 hrs, and then were stained with anti-MANF (red) and anti-p65 (green) antibodies. DAPI was used to stain the nuclei (blue). Scale bar = 10 µm. (c) Co-localization of MANF and p65 in the synovial tissue. The synovial tissues isolated from AIA rabbits were stained with anti-MANF (green) and anti-p65 (red) antibodies, respectively. DAPI was used to stain the nuclei (blue). Scale bar is 50 µm (upper panel) and 20 µm (lower panel), respectively. (d) Verifying the interaction of MANF and p65 by co-immunoprecipitation. The cells treated with TM (2.5 μg/ml) for 12 hrs were lysed for immunoprecipitation with an anti-p65 antibody, followed by Western blotting with the indicated antibodies. (e) MANF interacts with p65 in the nuclei. The cells were transfected with MANF-GFP, and then treated with or without TM (2.5 μg/ml) for 12 hrs. The cytosolic and nuclear fractions were immunoprecipitated with an anti-p65 antibody. Tubulin and histone H3 were used as the markers of the cytosol and nucleus, respectively. The experiments in all the panels were repeated for at least three times.

To explore the potential function of MANF in nuclei, we screened the MANF-interacting proteins using a yeast two-hybrid system. A human fetal cDNA library was screened with MANF as the bait. Surprisingly, p65, a subunit of NF-κB, was found among the positive clones. To verify the interaction between p65 and MANF, we co-transformed AH109 yeast competent cells with pGADT7-p65 and pGBKT7-MANF. As shown in Figure 3a, MANF interacted with p65. Using immunofluorescent analysis, we observed the co-localization of MANF and p65 in the nuclei in the primarily cultured FLS from the normal synovium after TM or LPS treatment (Fig. 3b). In the synovium of AIA rabbits, we also observed the co-localization of MANF and p65 in the nuclei of FLS (Fig. 3c).

To investigate whether MANF forms a complex with p65 under ER stress, the cells were treated with TM (2.5 μg/ml) for 12 hrs, and the full lysate was used for immunoprecipitation by using p65 antibody. Isotype IgG was used as the control. We found that endogenous MANF interacted with p65 only with exposure to TM (Fig. 3d). To detect the subcellular fraction of the interaction, we over-expressed MANF-GFP in the cells and treated the cells with TM (2.5 μg/ml) for 12 hrs. The cytosolic and nuclear fractions were isolated and processed for immunoprecipitation with the anti-p65 antibody. The result showed that MANF associated with p65 in the nuclear fraction, which occurred in the presence of TM (Fig. 3e). However, the interaction of cytoplasmic p65 with MANF was not detectable with or without TM treatment (Fig. 3e). Therefore, these results indicate that MANF is associated with p65 in the nuclei under the condition of ER stress.

### MANF binds to the DNA binding domain of p65 through its C-terminal SAP-like domain

**Figure 4:**
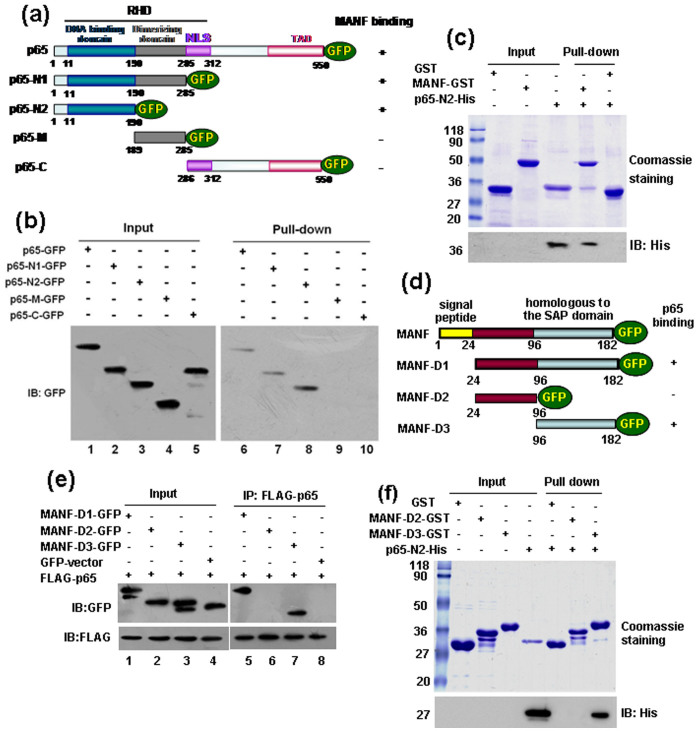
MANF binds to the DNA binding domain of p65 through its C-terminal SAP-like domain. (a) Schematic illustration of p65 and its truncates. RHD, Rel homology domain; NLS, nuclear localization signal; TAD, transactivation domain. (b) Mapping the domain of p65 binding to MANF by semi-pull-down assay. MANF-GST bound to glutathione beads was used as an affinity matrix to absorb full-length GFP-tagged p65 and its truncates expressing in 293T cells. The proteins were blotted with an anti-GFP antibody. (c) Confirmation of the binding site of p65 and MANF by *in vitro* pull-down assay. MANF-GST bound to glutathione beads were used as an affinity matrix for absorbing 6 × His-tagged p65-N2 fragment. The SDS-PAGE gel was stained with Coomassie brilliant blue (upper panel) and then blotted with an anti-His antibody (lower panel). (d) Schematic illustration of MANF and its truncates. (e) Mapping the binding site of MANF and p65 by co-immunoprecipitation. 293T cells were co-transfected as indicated, and treated with tunicamycin for 12 hrs at 24 hrs after transfection. (f) Confirming the binding site of MANF and p65 by pull-down assay. MANF-GST bound to glutathione beads were used as an affinity matrix for absorbing His-tagged p65-N2 fragment. The SDS-PAGE gel was stained with Coomassie brilliant blue (upper panel) and then blotted with anti-His antibody (lower panel). The experiments in panel b-c and e-f were repeated for at least three times.

To explore the MANF-binding region within p65, we constructed p65 truncates (Fig. 4a). We found that the lack of amino acids 1-190 at the N terminus of p65 resulted in its complete inability to interact with MANF (Fig. 4b, lane 9–10). By contrast, the fragment 1-190 at the N terminus of p65 (p65-N2) interacted with MANF (Fig. 4b, lane 8). Therefore, the results suggest that p65 binds to MANF via its DNA binding domain (amino acids 1-190). To further confirm whether MANF directly interacts with p65, we performed an *in vitro* GST pull-down assay using recombinant MANF-GST and p65-N2-His (amino acids 1-190) expressed in *E. coli.* As shown in Figure 4c, MANF-GST, but not GST, was able to pull down p65-N2-His, demonstrating that MANF physically binds to the DNA binding domain of p65. Similarly, we constructed MANF truncations to explore the p65-binding region (Fig. 4d). The plasmids were transfected into the cells and treated with TM for 12 hrs. We found that the lack of amino acids 96-182 at the C terminus of MANF eliminated its ability to interact with p65 (Fig. 4e, lane 6). A GST pull-down assay also showed that MANF-D3-GST including the C-terminal SAP-like domain (amino acids 96-182) was able to pull down p65-N2-His (Fig. 4f). These results suggest that MANF binds to the DNA-binding domain of p65 through its C-terminal SAP-like domain.

### MANF suppresses TNF-α-triggered NF-κB activation

**Figure 5:**
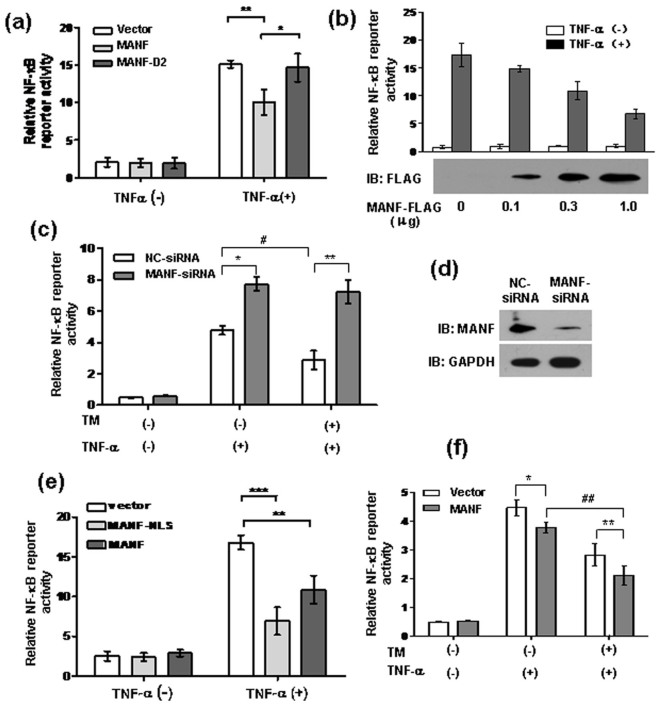
MANF suppresses TNF-α-triggered NF-κB activation. (a) MANF over-expression repressed TNF-α-induced NF-κB activity. 293T cells were transiently co-transfected with κB-Luciferase and MANF-FLAG or MANF-D2-FLAG plasmids for 24 hrs and treated with TNF-α (10 ng/ml) for 8 hrs. The data are the means ± SD of at least three independent experiments. * P = 0.0354, ** P = 0.0077, compared with the vector control. (b) The dose-dependent effect of MANF on TNF-α-induced NF-κB activity. 293T cells were co-transfected with κB-Luciferase reporter plasmid and the different amounts of MANF-FLAG. At 24 hrs after transfection, the cells were treated with TNF-α (10 ng/ml) for 8 hrs. MANF expression was confirmed by Western blotting with an anti-FLAG antibody (lower panel). (c) MANF knockdown with siRNA increased TNF-α-induced NF-κB activity. 293T cells were transfected with κB-Luciferase plus MANF-siRNA. The non-targeting siRNA (NC-siRNA) was used as the control. At 24 hrs after transfection, we pretreated the cells with TNF-α for 12 hrs. Then, the cells were exposed to TM for 6 hrs before harvesting and subjecting to a luciferase assay. The data are the means ± SD of at least three independent experiments. * P = 0.0014, ** P = 0.0013, compared with the NC-siRNA control; ^#^ P = 0.0075, compared with the cells untreated with TM. (d) The effectiveness of MANF-siRNA was monitored by Western blotting. 293T cells were transfected with MANF-siRNA and NC-siRNA for 36 hrs, following by Western blotting analysis. (e) MANF-NLS over-expression repressed TNF-α-induced NF-κB-Luciferase activity. NLS, nuclear localization signal. The data are the means ± SD of at least three independent experiments. ** P = 0.0068, *** P = 0.0010, compared with the vector control. (f) ER stress enhanced the suppression of MANF on the TNF-α-induce NF-κB activation. 293T cells were transiently co-transfected with κB-Luciferase and MANF-FLAG plasmids. At 24 hrs after transfection, we pretreated the cells with TNF-α for 12 hrs. Then, the cells were exposed to TM for 6 hrs before harvesting and subjecting to a luciferase assay. The data are the means ± SD of at least three independent experiments. * P = 0.0241, ** P = 0.0039, compared with vector control; *^##^* P* = *0.0016, compared with the cells untreated with TM.

We have mentioned above that MANF interacts with p65 in the nuclei. We wondered whether MANF affects NF-κB-mediated target genes activation. To address this issue, we transiently cotransfected a luciferase reporter construct containing three copies of the NF-κB binding site (3 × κB-Luc) together with MANF-FLAG or MANF-D2-FLAG, a truncate lacking p65-binding domain, into 293T cells. At 24 hrs post-transfection, the cells were treated with TNF-α (10 ng/ml) for 8 hrs. A luciferase reporter assay showed that MANF over-expression inhibited TNF-α-induced NF-κB activation (Fig. 5a). Meanwhile, lacking p65-binding domain at the C-terminus of MANF (MANF-D2) abolished the inhibition of NF-κB activation, suggesting a critical role for the SAP-like domain of MANF in inhibiting the NF-κB signaling (Fig. 5a). To confirm this result, 293T cells were transfected with different doses of MANF-FLAG plasmids. MANF-FLAG expression was detected with anti-FLAG antibody (Fig. 5b, lower panel). We found that TNF-α treatment led to a dramatic increase in the activity of κB-luciferase, which was inhibited by MANF in a dose-dependent manner (Fig. 5b). To further investigate the effect of MANF on NF-κB-mediated target gene activation, we used siRNA to knockdown endogenous MANF^20^, and the effectiveness of MANF-siRNA was confirmed by monitoring MANF protein levels (Fig. 5d). Knockdown of endogenous MANF significantly enhanced TNF-α-stimulated reporter gene activity (p = 0.0014) (Fig. 5c). To determine whether nuclear MANF regulates NF-κB reporter gene activity more effectively, we constructed FLAG-tagged MANF-NLS (including the nuclear localization signal: RRGGCGSVGRRRQRR at the N-terminus of MANF) and transfected it into the cells. The result revealed that MANF-NLS, compared with wild type MANF (p = 0.0068), inhibited TNF-α-induced NF-κB activation more significantly (p = 0.001) (Fig. 5e), suggesting that nuclear MANF predominantly attenuates NF-κB activity. These results suggest that MANF may be an inhibitor of NF-κB activity.

Several previous reports have shown that ER stress and consequent UPR induce a rapid and modest activation of NF-κB, whereas persistent UPR may inhibit NF-κB activation in the later phase^14^,^15^,^20^,^21^. MANF protein is induced in a time-dependent manner and is markedly increased at 6 hrs after TM treatment^20^; the later also shows an inhibitory effect on NF-κB activation as mentioned above. Therefore, we wondered whether MANF is involved in the inhibition of NF-κB activation mediated by ER stress. To address this issue, we pretreated the cells with TNF-α for 12 hrs at 24 hrs after transfection. The cells were exposed to TM for 6 hrs before harvesting and subjecting to a luciferase assay. The results showed that TM attenuated TNF-α-induced NF-κB activation, and knockdown of endogenous MANF abolished the suppressive effect of TM (Fig. 5c). Consistently, MANF over-expression enhanced the suppressive effect of TM on TNF-α-induced NF-κB activation (Fig. 5f). Therefore, we concluded that MANF was involved in the suppression of NF-κB activation induced by ER stress.

### MANF blocks p65 binding to its target genes

**Figure 6:**
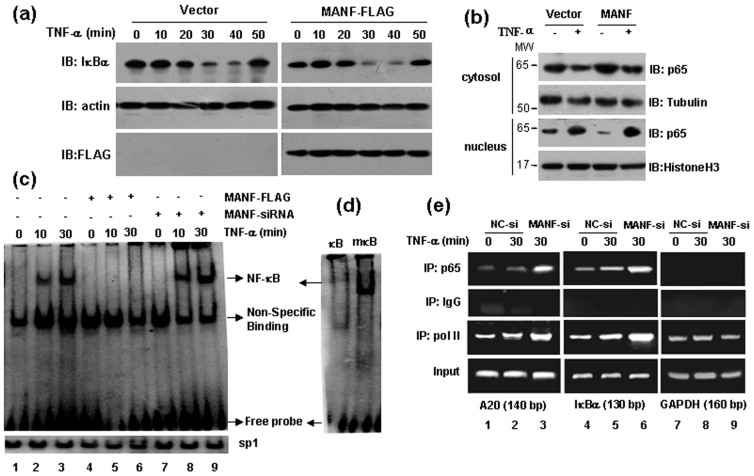
MANF represses the binding of p65 to its target genes. (a) MANF does not influence the total level of IκBα induced by TNF-α. 293T cells were transfected with MANF-FLAG and control vector, then treated with TNF-α (10 ng/ml) for the indicated times. Western-blotting was performed to check the level of IκBα. (b) MANF does not affect TNF-α-induced nuclear translocation of p65. 293T cells were transfected with MANF-GFP or GFP vector (as a control), and treated with TNF-α (10 ng/ml) for 30 min. Cytoplasmic and nuclear fractions were prepared and immunoblotted with the indicated antibodies, respectively. (c) MANF represses the DNA-binding activity of p65. The cells were transfected with the indicated plasmids for 36 hrs and treated with TNF-α for 10-30 min. Nuclear NF-κB was examined by EMSA. Sp1 was used as a loading control. (d) Competition analysis used in EMSA. 100-fold excess of unlabeled wild-type or mutant κB probes were added to the reaction mixtures. (e) MANF represses the association of p65 with its target promoters. 293T cells were transfected with MANF-siRNA for 36 hrs and treated with TNF-α for 30 min. ChIP assay was performed by using the indicated antibodies. NC-si, NC-siRNA; MANF-si, MANF-siRNA. The experiments in all the panels were repeated for at least three times.

To further clarify how MANF modulates NF-κB activity, we first questioned whether MANF affects the level of TNF-α-induced IκB-α and the subsequent nuclear translocation of NF-κB p65. To reach this goal, 293T cells were transfected with MANF-FLAG and treated with 10 ng/ml TNF-α for the indicated times in Figure 6a. The results revealed that MANF over-expression did not affect the total level of IκB-α (Fig. 6a). To observe p65 nuclear translocation, 293T cells were transfected with MANF-GFP, and treated with TNF-α for 30 min. The result showed that the level of p65 was changed neither in cytosol nor in nucleus after treatment with TNF-α and/or in presence of MANF. These results suggest that MANF affects neither IκB-α protein level or p65 nuclear translocation.

We have mentioned above that MANF bound to the DNA-binding domain of p65, which specifically happened inside the nucleus. Therefore, we went on to address whether this interaction affects p65 binding to its target DNA. We transfected 293T cells with MANF-FLAG or MANF-siRNA, and stimulated with TNF-α (10 ng/ml) for the indicated times (Fig. 6c). The nuclear extracts were prepared and then processed for electrophoretic mobility shift assay (EMSA). As expected, TNF-α induced endogenous nuclear p65 to bind to the probe strongly (Fig. 6c, lane 2–3). The amount of nuclear p65 binding to probe DNA (as indicated by retarded gel migration) was markedly diminished in the cells that were transfected with MANF-FLAG (Fig. 6c, lane 4–6), compared with the controls (Fig. 6c, lane 1–3). In contrast, the amount of nuclear p65 binding to probe DNA was increased in the cells that were transfected with MANF-siRNA (Fig. 6c, lane 7–9). The transcription factor sp1 as a negative control was expressed constitutively inside the nucleus and remained intact in response to MANF knockdown or over-expression (Figure 6c, lower panel). Additionally, we performed a ChIP assay to further examine the binding of p65 to the promoters of endogenous NF-κB target genes. Consistently, knockdown of the endogenous MANF resulted in considerable increases in p65 association with its cognate promoters (A20 and IκBα) upon TNF-α stimulation (Fig. 6e, lane 3, 6). However, MANF knockdown increased the binding of RNA polymerase II, one of the components of NF-κB transcriptional enhanceosome, to the promoters of A20 and IκBα. Reasonably, MANF knockdown had no effect on the glyceraldehyde-3-phosphate dehydrogenase (GAPDH) transcriptional complex (Fig. 6e, lane 9). These results indicated that MANF blocks the DNA binding activity of p65.

### MANF suppresses the expression levels of NF-κB target genes

**Figure 7:**
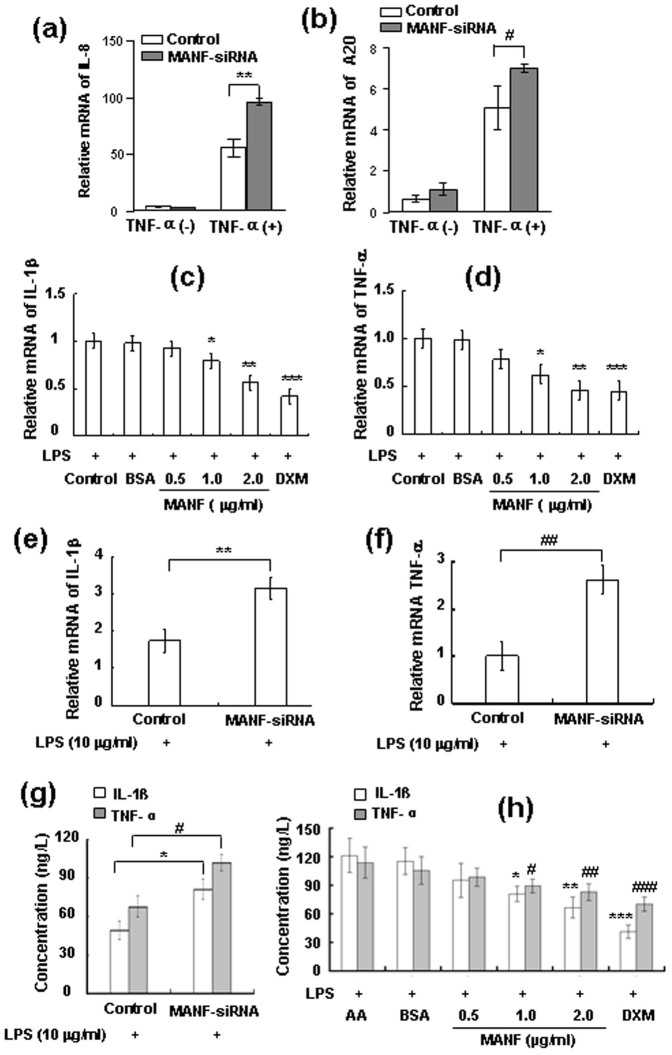
MANF suppresses the expressions of NF-κB target genes. (a–b) MANF knockdown increases the mRNA levels of IL-8 (a) and A20 (b). 293T cells transfected with MANF-siRNA were stimulated with TNF-α (10 ng/ml) for 30 min 24 hrs after transfection. Relative mRNA levels were analyzed by real-time qPCR. The data are expressed as the means ± SD of at least three independent experiments. ** P = 0.0079, ^# ^P = 0.0390, compared with the control. (c–d) Recombinant MANF inhibits the mRNA expressions of IL-1β (c) and TNF-α (d) in FLS. The FLS isolated from adjuvant arthritis (AA) rats were cultured in DMEM containing 10 μg/ml LPS and MANF protein for 24 hrs. BSA and DXM were used as the negative control and positive control, respectively. The mRNA levels were determined by RT-PCR. The data are expressed as the means ± SD of at least three independent experiments. In panel c, * P = 0.015, ** P = 0.002, *** P = 0.000, compared with BSA. In panel d, * P = 0.025, ** P = 0.001, *** P = 0.00, compared with BSA. (e–f) MANF-siRNA increases the expressional levels of IL-1β (e) and TNF-α (f) in FLS. The transfected FLS were treated with LPS (10 μg/ml) for 24 hrs. Relative mRNA levels were analyzed by RT-PCR. The data are the means ± SD of at least three independent experiments. ** P = 0.06, ^## ^P = 0.01, compared with siRNA control. (g) MANF-siRNA increases the secretion of IL-1β and TNF-α in FLS. The transfected FLS were treated with LPS (10 μg/ml) for 24 hrs. The levels of IL-1β and TNF-α were analyzed by ELISA. The data are expressed as the means ± SD from at least three independent experiments. * P = 0.036; ^# ^P = 0.017, compared with siRNA control. (h) Recombinant MANF decreases the secretion of IL-1β and TNF-α in FLS. The FLS of AA rats were incubated with 10 μg/ml LPS and MANF for 24 hrs. The levels of IL-1β and TNF-α in the medium were determined by ELISA. The data are expressed as the means ± SD from at least three independent experiments. * P = 0.018, ** P = 0.008, *** P = 0.000, ^# ^P = 0.03,^ ## ^P = 0.019, ^### ^P = 0.001, compared with BSA.

To investigate whether induction of NF-κB-dependent target genes was altered by MANF, 293T cells were transfected with control siRNA or MANF-siRNA and treated with TNF-α (10 ng/ml) for 30 min. Quantitative real-time PCR was performed to measure the expression of endogenous NF-κB target genes, such as IL-8 and A20. Consistently, the transcriptional levels of NF-κB target genes (IL-8 and A20) induced by TNF-α were increased when endogenous MANF expression was suppressed (Fig. 7a, b). To investigate the physiological significance of MANF up-regulation in inflammatory FLS, we expressed recombinant human MANF protein in *E.coli* and purified it. The FLS were isolated from the synovium of adjuvant arthritis (AA) rats and treated with LPS (10 mg/L) and different concentrations of MANF (0.5, 1.0, 2.0 μg/ml) for 24 hrs. The mRNA expressions of IL-1β and TNF-α in FLS were remarkably decreased after MANF treatment (2.0 μg/ml), compared with those treated with bovine serum albumin (BSA) (Fig. 7c, d). In this experiment, we also found that dexamethasone (DXM), as a positive control, inhibited IL-1β and TNF-α expression in FLS. To further confirm the inhibitory effect of MANF on the induction of inflammatory cytokines in FLS, we silenced the endogenous MANF with siRNA. The data showed that both IL-1β and TNF-α mRNA were significantly increased in FLS treated with LPS (10 μg/ml) after MANF-siRNA transfection (Fig. 7e, f). Meanwhile, recombinant human MANF protein decreased the secretion of IL-1β and TNF-α, whereas MANF-siRNA increased the levels of IL-1β and TNF-α in the medium of the cultured FLS (Fig. 7g, h). These results indicate that MANF suppresses the expressions of NF-κB target genes under the condition of inflammation.

### MANF inhibits the proliferation of synoviocytes isolated from adjuvant arthritis rats

**Figure 8:**
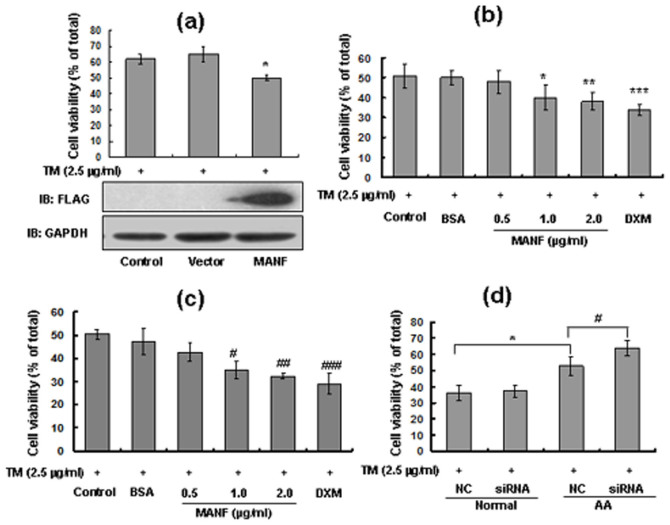
MANF suppresses the proliferation of inflammatory FLS. (a) MANF over-expression represses FLS proliferation. FLS were transfected with MANF-FLAG and treated with TM (2.5 μg/ml) for 24 hrs. FLS viability was determined using an MTT assay. MANF expression was detected by Western blotting. The data are the means ± SD of at least three independent experiments. * P = 0.03, compared with vector control. (b-c) Recombination MANF protein inhibits FLS proliferation. The FLS isolated from AA rats were cultured in DMEM medium containing 2.5 μg/ml TM and different concentrations of MANF protein (as indicated) for 24 hrs (b) and 48 hrs (c). BSA and DXM were used as negative control and positive control, respectively. FLS viability was determined using an MTT assay. The data are the means ± SD of at least three independent experiments. * P = 0.017, ** P = 0.008, *** P = 0.001, ^# ^P = 0.021,^ ## ^P = 0.003, ^### ^P = 0.007, compared with the BSA control. (d) MANF-siRNA promotes FLS proliferation. The normal and inflammatory FLS were transfected with MANF-siRNA and control siRNA for 24 hrs, then treated with TM (2.5 μg/ml) for 24 hrs. FLS viability was detected using an MTT assay. The data are the means ± SD of at least three independent experiments. * P = 0.041, ^# ^P = 0.011, compared with the siRNA controls.

To examine whether MANF affects the proliferation of synoviocytes, the FLS derived from AA rats were transiently transfected with MANF-FLAG plasmid. Twenty-four hours post-transfection, the cells were treated with 2.5 μg/ml TM for 24 hrs and cell viability was detected by MTT assay. The results showed that MANF over-expression inhibited FLS proliferation (Fig. 8a). To further confirm the inhibitory effect of MANF on FLS proliferation, FLS derived from AA rats were treated with different concentrations of recombinant human MANF and 2.5 μg/ml TM for 24 hrs, and then, the viability of synoviocytes was assayed by using an MTT assay. Compared with BSA, treatment with MANF (2.0 µg/ml) and DXM significantly inhibited the proliferation of FLS during ER stress (Fig. 8b). Furthermore, treatment with MANF (1.0 µg/ml) for 48 hrs produced the same inhibition effect on FLS proliferation (Fig. 8c), suggesting that MANF inhibits FLS proliferation during ER stress in a dose and time-dependent manner. Consistently, knockdown of endogenous MANF with siRNA promoted the proliferation of FLS isolated from AA rats, but not that from the normal rats after exposure to TM (2.5 μg/ml) for 24 hrs (Fig. 8d). These results suggest an inhibitory role for MANF in inflammatory cell proliferation, which support the notion that MANF plays an inhibitory role in the activation of NF-κB target genes.

## Discussion

Inflammation and ER stress are closely linked with each other. On the one hand, ER stress-induced UPR signaling is associated with the production of many pro-inflammatory molecules. All three main branches of the UPR (i.e., those based on the activities of the proteins PERK, IRE1a, and ATF6) have been shown to mediate pro-inflammatory transcriptional programs. NF-κB is one of the central mediators of pro-inflammatory pathways. Several molecular mechanisms have been proposed for the activation of NF-κB by ER stress. Tam AB *et al.* reported that IRE1-dependent homeostatic regulation on basal IκB kinase (IKK) activity is necessary for the effective activation of NF-κB by PERK. They found that combined inputs from both PERK and IRE1 are required for NF-κB activation during ER stress^22^. Hu *et al.* found that IRE1 forms a complex with IKK through TRAF2 under ER stress, and the kinase activity of IRE1 is essential for activating the IKK complex and the consequent phosphorylation and degradation of IκBα^8^. Some research has shown that the PERK-induced phosphorylation of eIF2α is essential for activation of NF-κB by ER stress. The authors identified that the repression of IκBα translation is important for NF-κB activation by eIF2α^10^. Similarly, the IRE1a–TRAF2 complex has also been shown to activate the JNK protein, which consequently phosphorylates and activates AP-1^13^. On the other hand, inflammatory stimuli or mediators may be responsible for the induction of ER stress. For example, GRP78 and CHOP are induced in the liver after the *in vivo* injection of IL-6 or IL-1β in mice. Splicing of XBP1 mRNA is also enhanced upon exposure to these cytokines^23^. TNF-α induces eIF2α phosphorylation and the consequent expression of GADD34 mRNA, induction of p50ATF6, and accumulation of the spliced forms of XBP1^24^. During inflammation, nitric oxide (NO) is abundantly produced by inducible nitric oxide synthase, and has the potential to induce ER stress^25^,^26^,^27^.

However, it has recently been reported that preceding ER stress responses may blunt the subsequent activation of NF-κB. Hayakawa K *et al.* reported that preceding ER stress causes insensitivity of mesangial cells to cytokine-induced activation of NF-κB via upregulation of A20 and downregulation of TRAF2^28^. They also found that ER stress depresses NF-κB activation in mesangial cells through the preferential induction of C/EBPβ^14^. Therefore, although ER stress triggers NF-κB activation in the early phase, it is possible that a subsequent UPR has the potential to inhibit NF-κB activation in a later phase. Beside these, some new mechanisms may be involved in the crosstalk between ER stress and NF-κB.

In this study, we demonstrated MANF, an ER stress inducible protein, was up-regulated by inflammation and conversely suppressed NF-κB signaling by interacting with the DNA binding domain of p65 through its C-terminal SAP-like domain. At first, we analyzed the mRNA expression of MANF in PBWC from RA and SLE patients. Compared with healthy controls, MANF mRNA was significantly up-regulated in these patients. This phenomenon was also observed in PBWC from rabbit AIA models. These data indicate that MANF might be involved in the regulation of inflammatory diseases. A recent result also showed that MANF inhibited oxygen-glucose deprivation-induced cell damage and inflammatory cytokine secretion in rat primary astrocytes^29^, which supports the notion of MANF in inflammation.

To further assess the expression characteristics of MANF in inflammatory tissue, we established rat and rabbit arthritis models and detected MANF in the two types of synoviocytes (MLS and FLS). We found that MANF was induced mainly in the FLS in the proliferative synovium, but very few in the MLS. Interestingly, although MANF was found predominantly in the cytoplasm, MANF occasionally appeared in the nuclei in some FLS that strongly expressed α-SMA. To confirm this finding and determine the factors that affect MANF translocation, we primarily cultured the FLS and treated them with LPS or TM. Interestingly, both the inflammatory-stimulator LPS and the ER stress inducer TM induced MANF re-localization to the nuclei, suggesting that inflammation and ER stress may mediate MANF re-localization.

MANF belongs to the family of neurotrophic factors. It protects neurons and repairs the Parkinson's disease-like symptoms in a rat 6-hydroxydopamine model^17^,^19^. MANF has also been identified as an ER stress responsive secretory protein and therefore, it should predominantly localize in the ER and Golgi in the cytoplasm. However, we found that MANF presented in the nuclei under ER stress and inflammation conditions. The unexpected pathophysiological behavior of MANF suggests that MANF may play an additional role in nucleus. To identify the nuclear targets of MANF, we employed a yeast two-hybrid assay to screen for MANF-interacting proteins. To our surprise, we found that p65, a subunit of NF-κB, strongly interacted with the C-terminal SAP-like domain of MANF. We also found the binding site was within the DNA binding domain of p65. In addition, our fractionation analysis revealed that MANF only interacted with p65 in the nucleus, but not the cytoplasm. This result suggests that p65 may be one of the intra-nuclear targets of MANF. Because MANF interacted with p65, it may be involved in the regulation of the NF-κB signaling pathway. Our data revealed that the recruitment of p65 to the promoters of NF-κB-regulated genes was significantly enhanced in MANF knockdown cells. Consistently, over-expression of nuclear accumulated MANF (MANF-NLS) markedly suppressed TNF-α-induced activation of NF-κB, and knockdown of endogenous MANF had the opposite effect, suggesting that MANF inhibited the transcriptional activity of NF-κB by blocking the DNA binding activity of NF-κB p65. Our results also indicate that recombinant MANF suppressed the expression of NF-κB target genes and inhibited the proliferation of inflammatory FLS. A recent study has revealed that the RTDL sequence in the C-terminus of MANF is necessary and sufficient for cell surface binding, involving an interaction with the KDEL receptors^30^. Therefore, we proposed that exogenous MANF might be a candidate acting as a ligand for KDEL receptors to exert its effect on FLS.

The prominent finding for MANF in this study is that the C-terminal SAP-like domain of MANF directly interacts with the DNA-binding domain of p65, which helps us to better understand the function and underlying mechanisms of MANF. Recent research shows that the C-terminal domain of MANF is homologous to the SAP domain of Ku70. The DNA repair protein Ku acts as a heterodimer of Ku70 and Ku80 subunits and binds to DNA ends or to single- to double-strand transition^31^,^32^,^33^. The SAP motif contains positively charged amino acids that might bind to the backbone of the DNA^34^,^35^,^36^. Therefore, we concluded that MANF competitively inhibits the binding of p65 to its cognate promoters through the C-terminal SAP-like domain, thus attenuating the transcriptional activity of NF-κB.

In present study, we demonstrated that MANF was elevated in the inflammatory diseases, and it conversely suppressed NF-κB activation and the production of pro-inflammatory factors in the primarily cultured FLS. According to these results, we proposed that the elevated levels of MANF must be required for maintaining the homeostasis during an inflammatory response. Inflammatory response causes ER stress. ER stress is an adaptive response at the early stage. However, ER stress and the consequent UPR facilitate the production of pro-inflammatory molecules, because activated MLS are resistant to ER stress-induced apoptosis^37^, which aggravate ER stress. The longevity and intensity of inflammation is detrimental to cells and tissues. In order to restrict inflammation, MANF may be induced as a compensatory mechanism. Therefore, we speculate that MANF induction governed by ER stress may aim to limit tissue damage and aid in tissue repair via inhibiting an inflammatory response. Taken together, our data indicate that MANF may be a novel negative regulator of inflammation that mediates the crosstalk between the NF-κB pathway and ER stress as well as can prevent the proliferation of inflamed cells.

## Methods

### Patients

The patients diagnosed with SLE and RA were recruited from the First Affiliated Hospital of Anhui Medical University and Xuzhou Central Hospital. The controls included age-matched healthy donors which were recruited from the First Affiliated Hospital of Anhui Medical University. All the methods in this study were carried out in accordance with the Helsinki criteria and were approved by the Ethics Committees of Anhui Medical University. Written informed consent was obtained from all patients and healthy donors. Sixty five SLE patients were selected after meeting at least 4 out of 11 American College of Rheumatology (ACR) criteria^38^. Lupus disease activity was scored according to the SLE disease activity index (SLEDAI)^39^. All except 6 SLE patients were treated with non-steroidal anti-inflammatory drugs and/or disease-modifying anti-rheumatic drugs. In total, 63 RA patients, with a mean age of 36 years (range from 19 to 79), were selected according to the ACR criteria, 54 were females and 9 were males. Disease activity in these patients was evaluated as the disease activity score in 28 joints (DAS28) according to Anderson et al.^40^. A total of 49 patients were positive for rheumatoid factor or anti-cyclic citrullinated peptide antibodies (CCP). The mean age of 69 healthy controls (NC) was 33 in the range of 18 to 58 years, where 18 donors were males and 51 females. The controls were those individuals without any medications or indications.

### Animals

Adult female New Zealand White rabbits weighing 2.0–2.5 kg and male Sprague-Dawley (SD) rats in a SPF grade (6–8 weeks of age, 180–220 g) were obtained from the Laboratory Animal Center of Anhui Medical University. All animals were fed with standard laboratory diet and tap water in a temperature- and humidity-controlled animal house under 12-h light-dark cycles. All the procedures were performed in accordance with the Guideline of Animal Care and Use Committee of Anhui Medical University and were approved by the Ethics Committees of Anhui Medical University.

### Reagents and plasmids

Antibodies against MANF (Abcam, Cambridge, Massachusetts, USA), α-SMA (Abcam, Cambridge, Massachusetts, USA), p65 (Abcam, Cambridge, Massachusetts, USA), IκB-α (Abcam, Cambridge, Massachusetts, USA), CD68 (AbD Serotec, Raleigh, North Carolina, USA), Alexa Fluor 488-conjugated and 568-conjugated IgG (Invitrogen, Carlsbad, California, USA) were used. The mouse anti-MANF antibody was produced in our laboratory^41^. For transient knockdown of MANF, siRNA1 [GGA CCU CAA AGA CAG AGA UTT (sense)], siRNA2 [GCA GAU CGA CCU GAG CAC ATT (sense)] and the negative control siRNA were purchased from Ambion (Austin, USA)^20^. p65 and its truncations were constructed by a PCR-based approach and cloned into mammalian or bacterial expression vectors as indicated. MANF and its truncates were constructed from pCIneo-MANF-FLAG, which was described previously^20^, based on a RT-PCR amplification. The PCR products were cloned into pEGFP-C2 and pGEX-6p-1 vector, respectively. For NLS-MANF plasmid, the PCR product was cloned into pEGFP-N1 vector. The details of cloning information were listed the supplemental data (Suppl. 1). All the constructs were subjected to sequencing for verification. The reporter genes (3 × κB-Luc and pFR-Luc) were kindly provided by Pro. Wancheng Li (University of Nebraska Medical Center, USA). The Enzyme Linked Immunosorbent Assay Kit for rats IL-1β and TNF-α detection were from R&D systems (Minneapolis, MN, USA).

### Induction of rabbit AIA

Rabbits were sensitized by intradermal injection of methylated bovine serum albumin (mBSA, Sigma; 0.5 ml containing 2 mg of mBSA) suspended in Complete Freund's adjuvant (FCA, Sigma) at 3-5 sites on day 0 and day 14. The controls were injected with saline solution. On day 28, the rabbits were anesthetized with methohexital (10 mg/kg, iv), and the right knee joints were intra-articularly injected with 1 mg of mBSA in 0.5 ml of 5% glucose solution, while the left knee joints were injected with 0.5 ml of saline solution as the sham-operation controls. The perimeter of the joints was measured with a caliper at regular intervals.

### Induction of rat adjuvant arthritis (AA)

The rats were immunized with FCA containing 10 mg of heat-inactive BCG in 1 ml of paraffin oil. On day 0, 0.1 ml of FCA was intradermally injected into the left hind paw of each rat, while the control rats received an equal volume of saline solution. On day 20, the synovium of AA rats was used.

### Immunohistochemistry

Immunohistochemistry was performed on sections as described previously^18^. Briefly, the paraffin sections were incubated with primary antibodies overnight at 4°C. After washing in PBS, the sections were incubated with the appropriate biotinylated secondary antibody for 1 hr at 37°C, which followed by incubation with horseradish peroxidase conjugated streptavidin for 15 min at 37°C. Immunostaining was developed by application of 3, 3′-diaminobenzidine tetrahydrochloride for 1–3 min. Then, the sections were counterstained with hematoxylin. Images were acquired using Olympus Microscope BX53 and cellSens Standard software.

### Synoviocytes culture and DNA transfection

Synoviocytes, isolated from the knee synoviums of normal or arthritis rats, were cultured in DMEM supplemented with 20% FBS in a humidified 5% CO_2_ atmosphere. In passage 3~9, the synoviocytes consisted of a homogeneous population of synovial fibroblasts and were used for experiments. DNA or siRNA was transfected into the synoviocytes using Lipofectamine 2000 (Invitrogen). The cells were transfected with 100 nM siRNA oligonucleotide for 36 hrs to knock down endogenous MANF. The non-targeting siRNA was used as a control.

### Yeast two-hybrid assay

MANF was fused to the GAL4 DNA-binding domain and p65 was fused to the GAL4 transactivation domain. Interactions were detected by the ability of cells to grow on medium lacking Ade, Trp, Leu, and His. Yeast cells co-transformed with pGBKT7-53 and pGADT7-T were used as the positive control. The negative controls were co-transformed with pGBKT7-lam and pGADT7-T.

### Luciferase assay

Cells were cotransfected with plasmids/siRNA and reporters as indicated. After transfection, the cells were treated with the indicated reagents. Luciferase activity was evaluated by the Dual-Luciferase Reporter Assay System (Promega, USA) according to the manufacturer's protocol. Experiments were performed in triplicates. Values obtained from Firefly luciferase signals were normalized to Renilla luciferase activity.

### Co-IP assay

Whole cell lysates were prepared 24 hrs post-transient transfection in a lysis buffer containing 20 mM Tris (pH 8.0), 138 mM NaCl, 10% glycerol, 1% Nonidet P-40, 10 mM NaF, 2 mM NaVO_4_, 1 mM pyrophosphoric acid and Complete^TM^ protease inhibitors (Roche Applied Science). The supernatant was collected and incubated with Protein A/G plus-agarose (Pierce) and relevant antibodies for 2 hrs at 4°C. The bound proteins were then eluted and subjected to Western blotting analysis.

### Pull-down assay

The N-terminal 190 aa of p65 was subcloned into pET28a vector (New England Biolabs, Beijing, China). The full length or truncates of MANF was subcloned into pGEX-6P1 vector (GE Healthcare, Wisconsin, USA). All constructs were sequenced and expressed in bacteria as fusion proteins. The purified soluble GST-fused MANF was pre-bound to glutathione–agarose (Sigma, USA). The column was washed using at least 5 × column volumes of buffer A (10 mM phosphate buffer, pH 7.4, 150 mM NaCl, 1% Triton X-100, and 1 mM PMSF), and then equilibrated with buffer B (50 mM Tris–HCl, pH 7.5, 100 mM NaCl, 0.1% Triton X-100, and 1 mM PMSF). The purified p65-N2-6 × His, which was dialyzed against buffer B, was loaded on the column and incubated for 1 hr at 4°C, and followed by washing with buffer B. The bound materials were then eluted with buffer C (50 mM Tris–HCl, pH 7.5, 500 mM NaCl, 0.1% Triton X-100, and 1 mM PMSF). The GST protein pre-bound column was used as negative control. All fractions collected were subjected to Western blotting analysis using an anti-His mAb (Cell Signal, Beverly, USA).

### Real-time qPCR

Total cellular RNA was isolated with TRIzol (Invitrogen) as instructed. Reverse transcription of purified RNA was performed using SuperScript III (Invitrogen). The quantification of gene transcripts was analyzed by real-time qPCR with SYBR green. Expression values were normalized to the level of β-actin mRNA. The primers used are listed as follows: MANF, forward 5′-TCA CAT TCT CAC CAG CCA CT-3′ and reverse 5′-CAG GTC GAT CTG CTT GTC ATA C-3′; IL-8, forward 5′-AGG TGC AGT TTT GCC AAG GA -3′ and reverse 5′-TTT CTG TGT TGG CGC AGT GT-3′; A20, forward 5′-GCG TTC AGG ACA CAG ACT TG-3′ and reverse 5′-GCA AAG CCC CGT TTC AAC AA-3′.

### RT-PCR

Total RNA was extracted from synovial tissue or synovial cells using Trizol reagent according to the manufacturer's protocol. Reverse transcription was performed using PrimeScript^TM^ RT Reagent Kit (Takara) at 37°C for 30 min. The primers used are listed as follows: MANF, forward 5′-TCC GCT ACT GTA AGC AAG GT-3′ and reverse 5′-CTT CAC CTA GGA TCT TGG TG-3′ ; IL-1β, forward 5′- TTG TGG CTG TGG AGA AGC TG-3′ and reverse 5′-GCC GTC TTT CAT ACA CAG G-3′; TNF-α, forward 5′-GCC AAT GGC ATG GAT CTC AAA G-3′ and reverse 5′-CAG AGC AAT GAC TCC AAA GT-3′; β-actin, forward 5′-TCA CCA ACT GGG ACG ACA T-3′ and reverse 5′-GCA CAG CCT GGA TAG CAA C-3′; Expression values were normalized to the level of β-actin mRNA, which was quantified by Bandscan software.

### Electrophoretic mobility shift assay (EMSA)

EMSAs were performed as described previously^42^. The probes used are as follows: wild-type κB probe (AGT TGA GGG GAC TTT CCC AGG C); mutant κB probe (AGT TGA GGC GAC TTT CCC AGG C); and sp1 probe (ATT CGA TCG GGG CGG GGC GAG C). Electrophoresis was performed on 6% non-denaturing Tris borate-EDTA-PAGE, and the gels were dried and subjected to autoradiography. For competition analysis, 100-fold excess of unlabeled wild-type or mutant κB probes were added to the reaction mixtures.

### Chromatin immunoprecipitation (ChIP) assay

293T cells were treated with 10 ng/ml TNF-α (R&D Systems, USA) for the indicated times prior to formaldehyde cross-linking. The ChIP assay was performed according to the ChIP protocol (Upstate). The following promoter-specific primers were used: human A20, forward 5′-CAG CCC GAC CCA GAG AGT CAC-3′ and reverse 5′-CGG GCT CCA AGC TCG CTT-3′; human GAPDH, forward 5′-AGC TCA GGC CTC AAG ACC TT-3′ and reverse 5′-AAG AAG ATG CGG CTG ACT GT-3′; and human IκBα, forward 5′-TAG TGG CTC ATC GCA GGG AG-3′ and reverse 5′-TCA GGC TCG GGG AAT TTC C-3′.

### Immunofluorescence staining

Synoviocytes grown on coverslips were treated with 10 μg/ml lipopolysaccharides (LPS, Sigma) for 48 hrs or 2.5 μg/ml tunicamycin (Sigma) for 16 hrs, and then fixed with 4% paraformaldehyde, permeabilized in 0.1% Triton X-100, blocked by 1% BSA, and stained with the indicated primary antibodies followed by fluorescent secondary antibodies. Nuclei were counterstained with DAPI (Sigma). Images were acquired using Olympus Microscope BX53/IX71and cellSens Standard software. Confocal images were acquired using confocal microscope(ZEISS LSM710)and ZEISS ZEN Imaging Software.

### MTT assay

Synoviocytes proliferation was assessed using MTT assay. Briefly, the cells were grown in 96-well plates. At the selected time points, MTT (500 µl; 5 mg/ml in PBS) was added to each well and incubated for 4 hrs at 37°C. MTT-formazan crystals were solubilized by DMSO. Absorbance of each well was measured at 570 nm. Experiments were repeated for three times, and the data are expressed as the mean ± SD of five wells.

### Statistical analyses

We used SPSS statistical software (version 16.0, SPSS, Chicago, IL). The data are expressed as the mean ± SD. Statistical comparisons were performed using a two-way ANOVA followed by the Scheffe's test. P<0.05 was considered statistically significant.

## Author Contributions

J.L. and Y.X.S. designed all the experiments; L.J.C., L.J.F., X.W., J.D., Y.C., W.Y., C.Y.Z., L.C., S.F. and Y.J.S. performed the experiments. L.J.C., J.D. and Y.X.S. wrote the manuscript. All authors reviewed the manuscript.

## Supplementary Material

Supplementary Information Supplemental information
