# Supplementary material for: Mesencephalic Astrocyte-Derived Neurotrophic Factor Is Involved in Inflammation by Negatively Regulating the NF-κB Pathway
Source: Sci Rep. 2015 Feb 2;5:8133. doi: 10.1038/srep08133 (PMC4313098; doi:10.1038/srep08133)
Supplement: Supplementary Information — Supplemental information [file srep08133-s1.pdf]

Supplemental information

Mesencephalic Astrocyte-Derived Neurotrophic factor Is Involved In Inflammation By Negatively Regulating the NF-κB Pathway

Lijian Chen, Lijie Feng, Xia Wang, Jian Du, Ying Chen, Wen Yang, Chengyue Zhou, Li Cheng, Yujun Shen, Shengyun Fang, Jun Li, and Yuxian Shen

Suppl. 1: Cloning information

Plasmid	Primer1	Primer2	Restriction Enzyme
p65-FLAG	CGGATCCAGATGGACGAACTGTT	CCTCGAGCTTAGGAGCTGATCTGACT	BamHI; XhoI
p65-His	CCTCGAGCATTAGGAGCTGATCTGACT	CGGATCCATGGACGAACTGTTC	XhoI; BamHI
pEGFP-p65-C	CAAGCTTCTTCCAGTACCTGCCAGATAC	CGGATCCTTAGGAGCTGATCTGACT	HindIII; BamHI
pEGFP-p65-N1	CAAGCTTCATGGACGAACTGTTC	CGGATCCTTCCATGGGCTCACTGA	HindIII; BamHI
pEGFP-p65-N2	CAAGCTTCATGGACGAACTGTTC	CGGATCCGTTGGGGGCACGATTGTCAAAGAT	HindIII; BamHI
pEGFP-p65-M	CAAGCTTCACTGCCGAGCTCAAGATCT	CGGATCCTTCCATGGGCTCACTGA	HindIII; BamHI
p65-AD	CGGATCCATATGGACGAACTGTTC	CCTCGAGTTAGGAGCTGATCTGACT	BamHI; XhoI
p65-C-AD	CGGATCCATTTCCAGTACCTGCCAGAT	CCTCGAGTTAGGAGCTGATCTGACT	BamHI; XhoI
p65-N1-AD	CGGATCCATATGGACGAACTGTT	CCTCGAGTTCCATGGGCTCACTGA	BamHI; XhoI
p65-N2-AD	CGGATCCATATGGACGAACTGTT	CCTCGAGGTTGGGGGCACGATTGTCAAAGAT	BamHI; XhoI
p65-M-AD	CGGATCCATACTGCCGAGCTCAAGATCT	CCTCGAGTTCCATGGGCTCACTGA	BamHI; XhoI
p65-M-His	CGGATCCACTGCCGAGCTCAAGATCT	CAAGCTTTTCCATGGGCTCACTGAG	BamHI; HindIII
MANF-DS-BD	GGAATTCATGGACTGCGAAGTTTGTATTTCTTATCTG	CGGATCCGTCTACAAATCGGTCCGTGCACT	EcoRI; BamHI
NLS-MANF-pEGFP-N1	C CTCGAG ATG GGG AAG TGG CAT GT	G GAATTC TG CAA ATC GGT CGG TGC	XhoI; EcoRI
pEGFP-MANF	GGAATTCATGAGGAGGATGAGGAG	CGGATCCCTACAAATCGGTCCGT	EcoRI; BamHI
pEGFP-MANF-D1	CGAATTCGCGCTGCGGCCGGGCGACT	CGGGATCCCTACAAATCGGTCCGTGCACT	EcoRI; BamHI
pEGFP-MANF-D2	CGAATTCGCGCTGCGGCCGGGCGACT	CGGGATCCCAGAGGCTTTGATACCTC	EcoRI; BamHI
pEGFP-MANF-D3	CGAATTCCTGGCCCACCACATCCCT	CGGGATCCCTACAAATCGGTCCGTGCACT	EcoRI; BamHI
MANF-D2-pGEX-6p-1	CGGATCCGCGCTGCGGCCGGGCGACT	CGGAATTCCAGAGGCTTTGATACCTC	BamHI; EcoRI
MANF-D3-pGEX-6p-1	CGGATCCCTGGCCCACCACATCCCT	CGGAATTCCTACAAATCGGTCCGTGCACT	BamHI; EcoRI
